# Simultaneous multiple single nucleotide polymorphism detection based on click chemistry combined with DNA-encoded probes†
†Electronic supplementary information (ESI) available. See DOI: 10.1039/c8sc00307f


**DOI:** 10.1039/c8sc00307f

**Published:** 2018-02-22

**Authors:** Qian-Yu Zhou, Fang Yuan, Xiao-Hui Zhang, Ying-Lin Zhou, Xin-Xiang Zhang

**Affiliations:** a Beijing National Laboratory for Molecular Sciences (BNLMS) , MOE Key Laboratory of Bioorganic Chemistry and Molecular Engineering , College of Chemistry , Peking University , Beijing 100871 , China . Email: zhouyl@pku.edu.cn ; Email: zxx@pku.edu.cn ; Fax: +86-10-62754112 ; Tel: +86-10-62754112

## Abstract

Single nucleotide polymorphisms (SNPs) are emerging as important biomarkers for disease diagnosis, prognostics and disease pathogenesis. As one type of disease is always connected to several SNP sites, there is great demand for a reliable multiple SNP detection method. Herein, we mimicked a ligation reaction based on DNA ligase and originally utilized an enzyme-free DNA template-directed click reaction for SNP detection. With 5′-alkyne and 3′-azide groups labelled on two oligonucleotide probes, the target DNA-directed Cu(i)-catalyzed alkyne–azide cycloaddition (CuAAC) click reaction produced a new DNA strand with a triazole backbone, as a mimic of a DNA phosphodiester linkage. Trace amounts of the target (as low as 25 fmol in 50 μL) could be sensitively detected using capillary gel electrophoresis with laser-induced fluorescence (CGE-LIF). Meanwhile, SNP caused an obvious difference in the efficiency of the click reaction, and 0.5% SNP could be easily detected. More importantly, multiplexed SNP detection in a one tube reaction was successfully achieved only by encoding different lengths of the DNA probes for the different SNP sites.

## Abstract 2

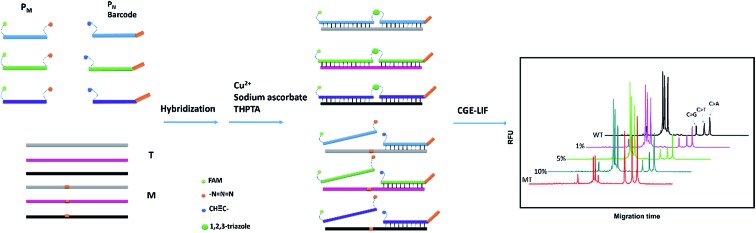
A novel strategy utilizing a DNA template-directed CuAAC click reaction to mimic a ligation reaction based on DNA ligase was successfully established for multiple SNP detection with high sensitivity and specificity.

## Introduction

Single nucleotide polymorphism (SNP) refers to a single base variation in the genomic sequence.^1^,^2^ It consists of single-nucleotide substitutions, insertions and deletions. It is the most common variant of human genetic variation.^3^ The detection of SNP is important for disease diagnosis, prognostics and disease pathogenesis.^4^,^5^ A series of various technologies have been developed for SNP genotyping.^6^–^12^ Among these methods, a ligation-dependent method performs as a highly sensitive and selective platform for the detection of SNP based on the specificity of the DNA ligase.^13^–^16^ However, enzymatic techniques usually require a strict environment and the operation is complex, which might hinder their use in clinical diagnosis, especially at point-of-care detection. Recently, non-enzymatic template-directed chemical reactions,^17^–^20^ such as nucleophilic substitution, cycloaddition and condensation, have shown great potential in the detection of DNA and RNA. Without the use of an enzyme, these methods can be used for the direct detection of nucleic acids in a complex matrix without sample preparation or target isolation.^21^,^22^ Furthermore, enzymatic methods cannot work in intact cells since it is difficult to deliver enzymes into cells, while these non-enzymatic approaches are robust and effective for their use in cells.^23^,^24^

Click chemistry^25^–^27^ is a simple and rapid synthetic method based on the carbon–heteroatom bond. The best example of click chemistry is the Cu(i)-catalyzed alkyne–azide cycloaddition (CuAAC) reaction. This reaction has the virtues of fast reaction speed, high yield of product and mild reaction conditions. Therefore, it is widely used in many fields such as proteomics,^28^ surface modification^29^ and biomedicine.^30^ Much research about oligonucleotide labelling using the CuAAC reaction has been reported.^31^–^35^ Brown *et al.* applied the CuAAC reaction to synthesize a covalently closed ssDNA circle and a dsDNA pseudohexagon, thereby constructing DNA nanotechnology.^31^ They then synthesized very long oligonucleotides using the CuAAC reaction and demonstrated that the artificial linkage was still functional in bacterial and human cells.^32^,^36^ With these advantages, the CuAAC reaction might be an excellent chemical ligation strategy to simulate the action of DNA ligase.

Generally, one type of disease is always connected to several SNP sites.^37^ Lung cancer is associated with multiple genes such as EGFR, ALK, MET and so on.^38^ The simultaneous multiple SNP detection for one type of disease can not only improve the accuracy of the diagnosis, but also provide some guidance for individualized targeted therapy. With the increasing attention on genome-wide linkage studies, more research focuses on the identification of the genetic variants related to complex diseases and traits.^39^,^40^ On this basis the construction of multiplex SNP genotyping methods can be an efficient and applicable approach for the detection of genes associated with the occurrence, development and treatment of disease.^41^,^42^ Meanwhile, the multiplex detection of SNPs can not only reduce the cost of genotyping, but also avoid tedious repeat operations. However, except DNA sequencing,^43^,^44^ most of the developed techniques for SNP detection lack multiplex detection abilities. DNA sequencing methods suffer from time-consuming procedures and expensive costs. Some fluorescence-based techniques^45^,^46^ have been developed for multiplex SNP detection, but their high-throughput is limited by the number of distinct fluorescent reporters, and spectral overlap cannot be avoided. Mass spectrometry based methods^47^,^48^ are limited by expensive instruments and the difficulty of the use of large-scale equipment for clinical testing. Capillary electrophoresis (CE)^49^–^51^ has been used for the multiplex detection of nucleic acids. The capability of CE for multiplex detection is related to its highly effective separation ability, so it can easily achieve real high-throughput detection.

Herein we have proposed a novel strategy for the first SNP discrimination based on CuAAC click chemistry combined with capillary gel electrophoresis with laser-induced fluorescence detection (CGE-LIF). Because of the high sequence specificity of the chemical reaction, SNP can be easily detected through the efficiency of the click reaction. Moreover, by encoding different lengths for the DNA probes for the different SNP sites, the ligated products produced by the CuAAC reaction can be simply separated using CGE. Therefore, multiplexed SNP detection in a one tube reaction can be easily achieved.

## Results and discussion

### The principle of CuAAC-based multiplexed SNP detection

**Scheme 1:**
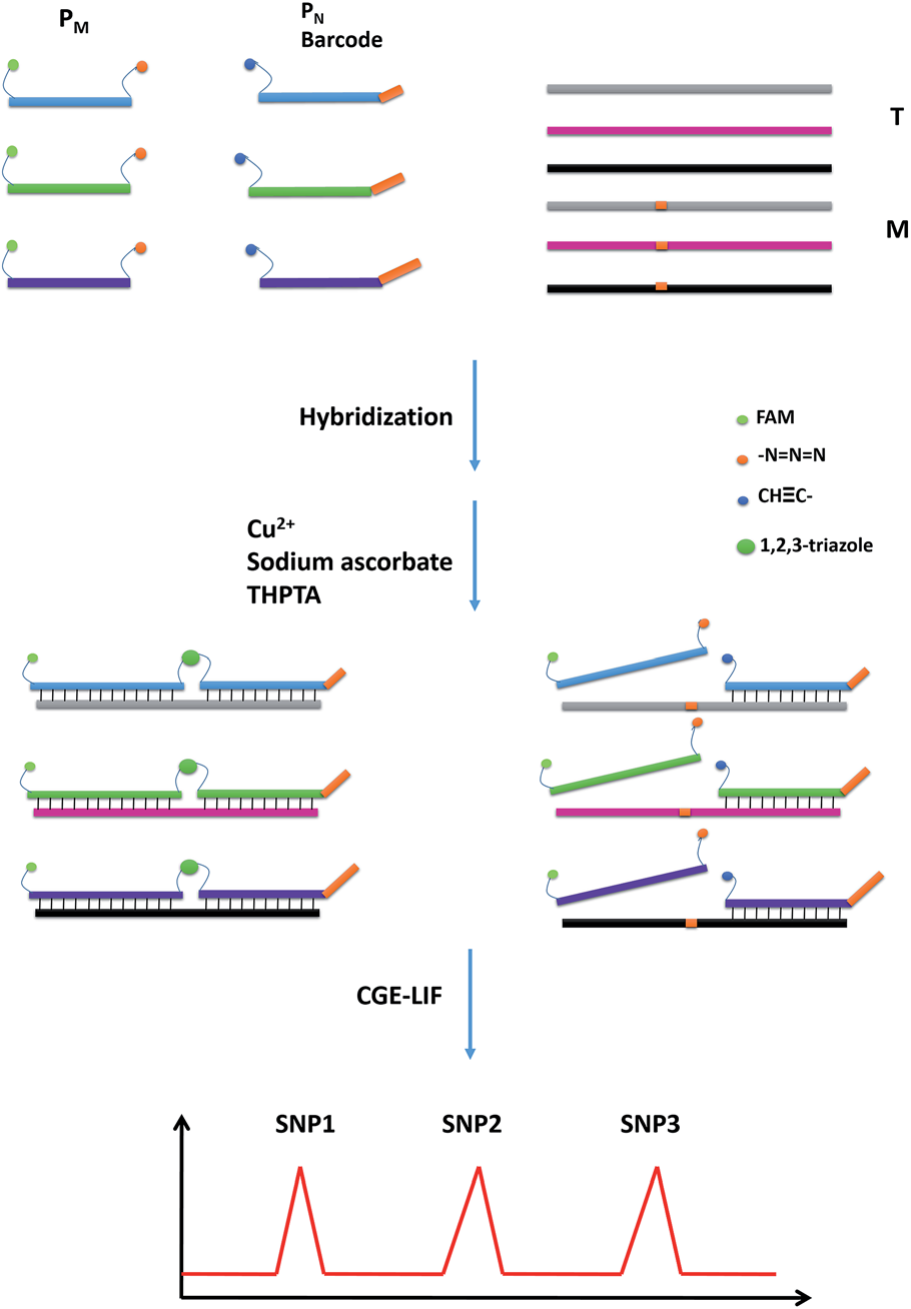
The schematic principle of CuAAC-based multiplexed SNP detection.

The design principle for the detection of multiplexed SNP is illustrated in Scheme 1. The probes P_M_ and P_N_ are designed to hybridize to a DNA target, wherein the probe P_M_ is modified with a fluorescent group FAM at the 5′ end and an azide group at the 3′ end, while an alkynyl group is modified at the 5′ end of the probe P_N_. Due to the relatively low concentrations of the probes, the ligation reaction caused by the free collision of two groups on the probes does not proceed easily in the absence of a perfectly matched target (T). However, in the presence of T, the two probes are induced to approach each other through hybridizing with T, and can be easily ligated through the CuAAC reaction. A single-base mismatched target (M) leads to a thermodynamic difference between the probe strands and M, which inhibits the formation of stable hybrid double chains. Due to the steric effects of these reactive groups, little ligation occurs. Furthermore, through the design of different lengths for the probes, the CuAAC reactions will yield FAM-labeled DNA strands with different lengths for the different SNP sites. The products can be easily separated using CGE and fluorophore labeled DNA can be detected by the LIF detection. Hence, multiplex SNP detection can be easily realized in a one-tube CuAAC reaction.

### The feasibility of the CuAAC-based SNP assay

**Fig. 1:**
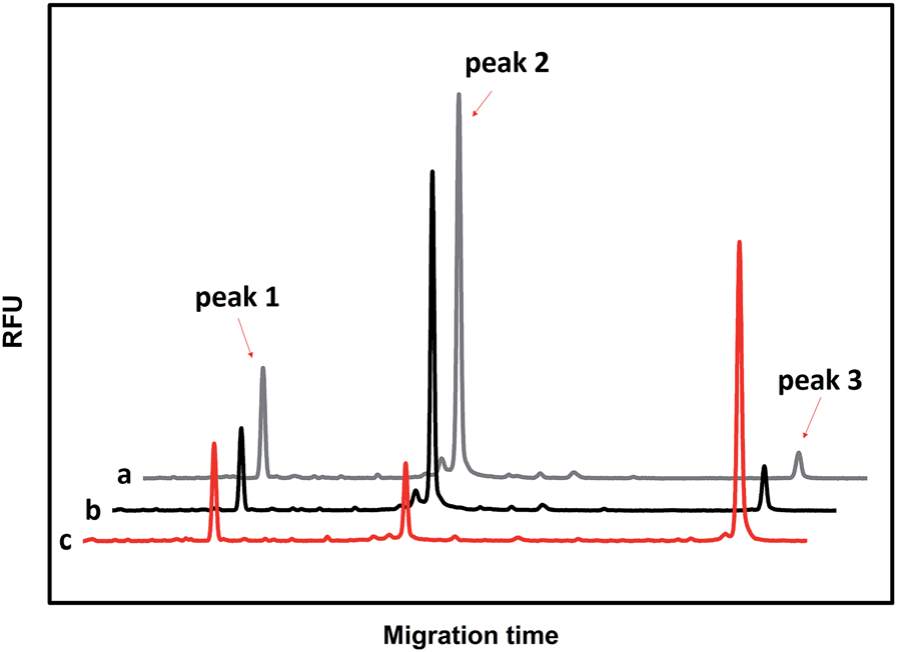
Electropherograms for the feasibility of CuAAC-based SNP detection. (a) 50 nM P_M_ + 50 nM P_N_; (b) 50 nM P_M_ + 50 nM P_N_ +100 nM M3; (c) 50 nM P_M_ + 50 nM P_N_ +100 nM T.

The performances of the probes P_M_ and P_N_ in the absence of T and in the presence of T and M3 (the sequences are illustrated in Table S1†) were investigated using CGE-LIF. As shown in Fig. 1, there are three peaks in the electropherograms. The retention time is related to the length of the oligonucleotide. The longer the length of the oligonucleotide, the later the retention time is. Peak 1 is attributed to an internal standard with a length of four bases, which is used to correct the peak areas of the products caused by an uncertainty of the sample injection amount. Peak 2 is attributed to unreacted P_M_. Peak 3 corresponds to the product of the click reaction between P_M_ and P_N_. In the absence of the DNA target, peak 3 is very small (Fig. 1a), indicating that the efficiency of the click reaction is quite low for the free collision of the probes P_M_ and P_N_ in solution. When comparing the electropherograms b and c with a, it can be seen that the addition of M3 only causes a small amount of the probes to be connected, while the addition of T can yield a large number of ligation products due to the stability of the duplex among P_M_, P_N_ and the target. HPLC-ESI-MS was conducted to further verify the formation of the ligation products. As shown in Fig. S1,† in the absence of T or in the presence of M3, we could only find peaks corresponding to P_M_ and P_N_ according to their molecular weight, while in the presence of T another peak belonging to the ligation product appeared, which indicated the successful ligation between P_M_ and P_N_ directed by T. Therefore, the assay has a good ability to discriminate between T and M, indicating that it can be used to detect the SNPs.

### Optimization of the experimental conditions

**Scheme 2:**
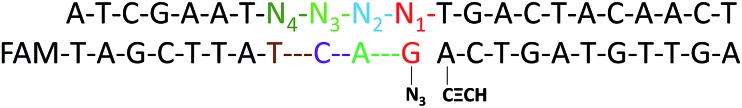
An image of DNA-directed reactions in the presence of a single-base mutation at the different positions N_1_ to N_4_.

In order to obtain optimal conditions for the CuAAC-based SNP assays, we investigated several reaction conditions which might influence the SNP discrimination ability. To utilize the sequence specificity of the DNA strands, an important factor is the discrimination ability to identify SNP at different positions relative to the template-directed click reaction site. Different DNA templates (Table S1†) with a single-base mutation at the different positions N_1_ to N_4_ (denoted as M1–M7) (Scheme 2) were designed. Since the type of base-mismatch has a big influence on SNP discrimination,^52^ to compare the site of the mismatch on the SNP discrimination ability, two different sites were compared by adjusting the base type of N_*x*_ and making the base pairs of the two mutation sites the same. As shown in Table S3,† the results for the SNP discrimination based on a target-directed click reaction demonstrate site-dependent effects. By calculating the relative peak area observed with the N_*x*_-mismatched strands of the four sites, substitution of the base at the N_3_ position resulted in the lowest efficiency of the click reaction. Therefore, single-base mutation at the N_3_ position holds great potential for the detection of SNP.

The effects of temperature on the connection efficiency of the CuAAC reaction caused by T and M3 were also investigated (using G > C substitution at the N_3_ position as a model). The melting temperatures (*T*_m_) of the probes P_M_ and P_N_ were found to be 25 °C and 29.1 °C, respectively. As shown in Fig. S2,† it was found that the connection efficiency for both T and M3 decreases with the increase of the temperature, indicating that the stability of the duplex between P_M_, P_N_ and the target is related to the reaction temperature. The best selectivity for SNP was achieved when the reaction was performed at 30 °C, which is slightly higher than the *T*_m_ of the probes.

The incremental ratio of the fluorescence intensity for the CuAAC product in the presence of T relative to that with M3 is plotted against the reaction probe ratio (P_M_ : P_N_) and the reaction time (Fig. S3 and S4†). When the amount of P_N_ gradually increases, the degree of discrimination becomes better. It is possible that the probe P_M_, which has a mutation base, is unstable for M3 at the reaction temperature, while the probe P_N_ is stable for T or M3. So if the amount of the probe P_N_ increases, there are more P_N_ chains that can be hybridized to the small amount of T. In this case, the probes P_M_ and P_N_ are more likely to be ligated with chemical reaction in the presence of P_M_. Finally, the probe reaction ratio of P_M_ : P_N_ = 1 : 10 was used as the reaction condition. The reaction time is another important factor for SNP detection. As shown in Fig. S4,† the discrimination effect is best at the reaction time of 30 min.

### Analytical performance of the CuAAC-based assay

**Fig. 2:**
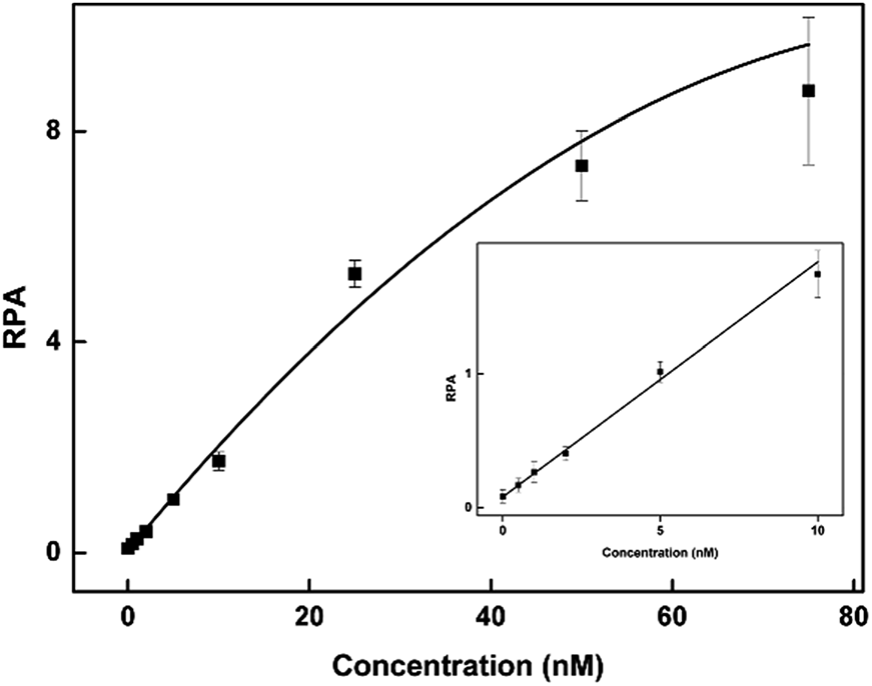
The corresponding calibration plot of RPA *vs.* the concentration of T. The inset is the linear part of the plot of RPA *vs.* the concentration of T. The concentration of T ranges from 500 pM to 10 nM. The standard deviation of three parallel experiments determined the error bar.

To evaluate the sensitivity of the CuAAC-based assay, we used T as the model. As demonstrated in Fig. 2, as the concentration of T increases, the fluorescence of the CuAAC products increases. There is a good linear relationship between the RPA and the T concentration ranging from 500 pM to 10 nM (RPA = 0.17 × *c*_T_ +0.093, *R*^2^ = 0.99). When the amount of T is as low as 25 fmol in 50 μL, it can still be sensitively detected, indicating that the CuAAC-based assay can be used for the quantification of the DNA target.

**Fig. 3:**
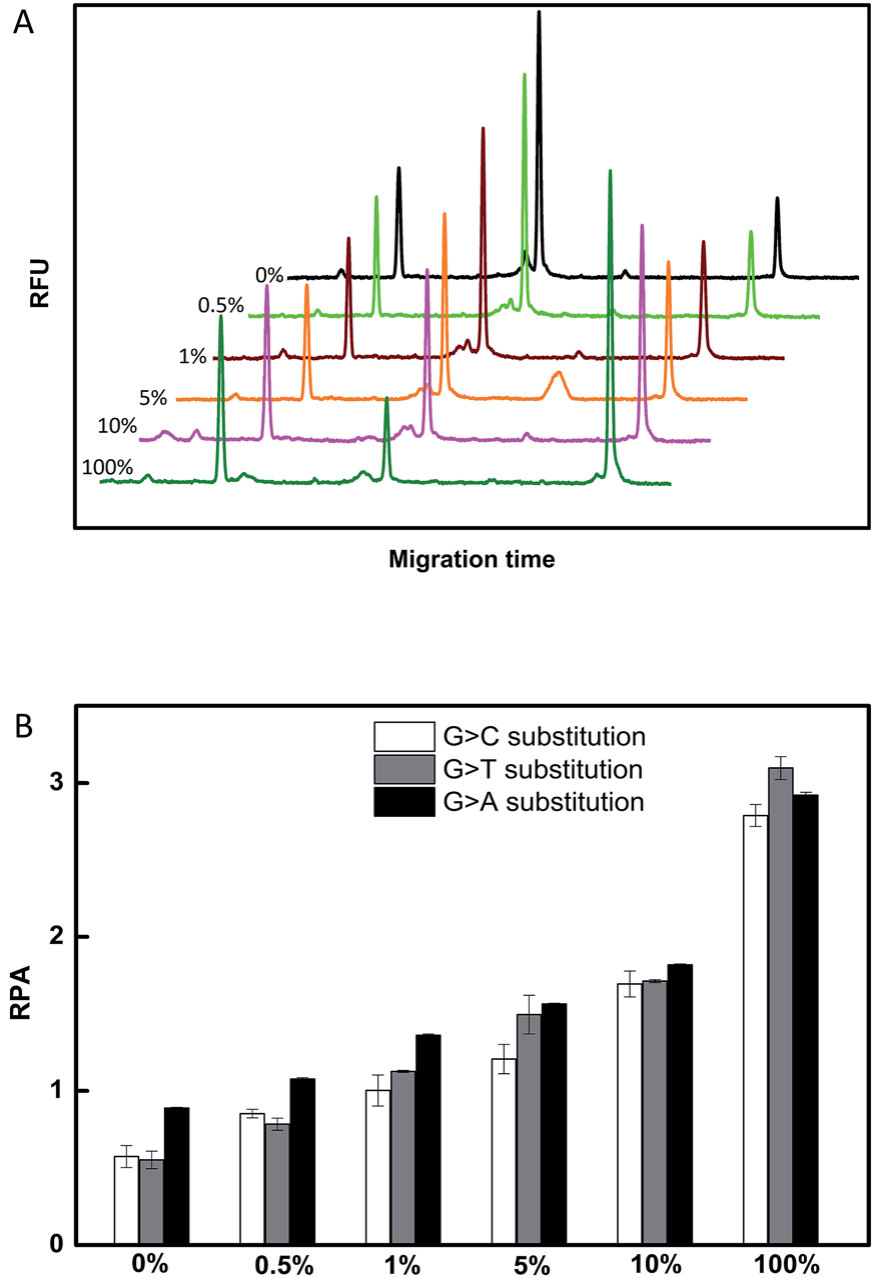
(A) The electropherograms for the detection of different abundances of T with M3 (G > C substitution). (B) The histogram of the CuAAC-based assay for different abundances of T with M3 (G > C substitution), M4 (G > T substitution), and M5 (G > A substitution). The standard deviation of three parallel experiments determined the error bar.

To investigate the selectivity of the assay for SNP detection, we applied this method to detect a low abundance of T in the presence of different amounts of different mismatched targets M. As demonstrated in Table S1,† we used the DNA probes to detect different base-mismatched types at the site N_3_. T was mixed with different mismatched targets M (M3, M4 or M5) at abundances of 0%, 0.5%, 1%, 5%, 10% and 100%. The sample mixtures were detected by the CuAAC-based assay using P_M_ and P_N_ as probes. The electropherograms for the detection of the C : C mismatched target in the probe/target hybrids (G > C substitution) is shown in Fig. 3A. The data shown in Fig. 3B were converted from the data shown in Fig. 3A by calculation of the fluorescence. As demonstrated in Fig. 3A and B, T can be obviously identified at an abundance of as low as 0.5% in the presence of a large amount of M3. Similarly, as low as 0.5% of the C : A and C : T mismatches in the probe/target hybrids can also be clearly detected using the CuAAC-based assay (Fig. 3B).

### Multiplexed SNP detection

Since SNPs have been used as molecular markers in clinical diagnosis and pharmacogenomic studies, the rapid, automated, accurate and affordable detection of SNPs is important.

**Fig. 4:**
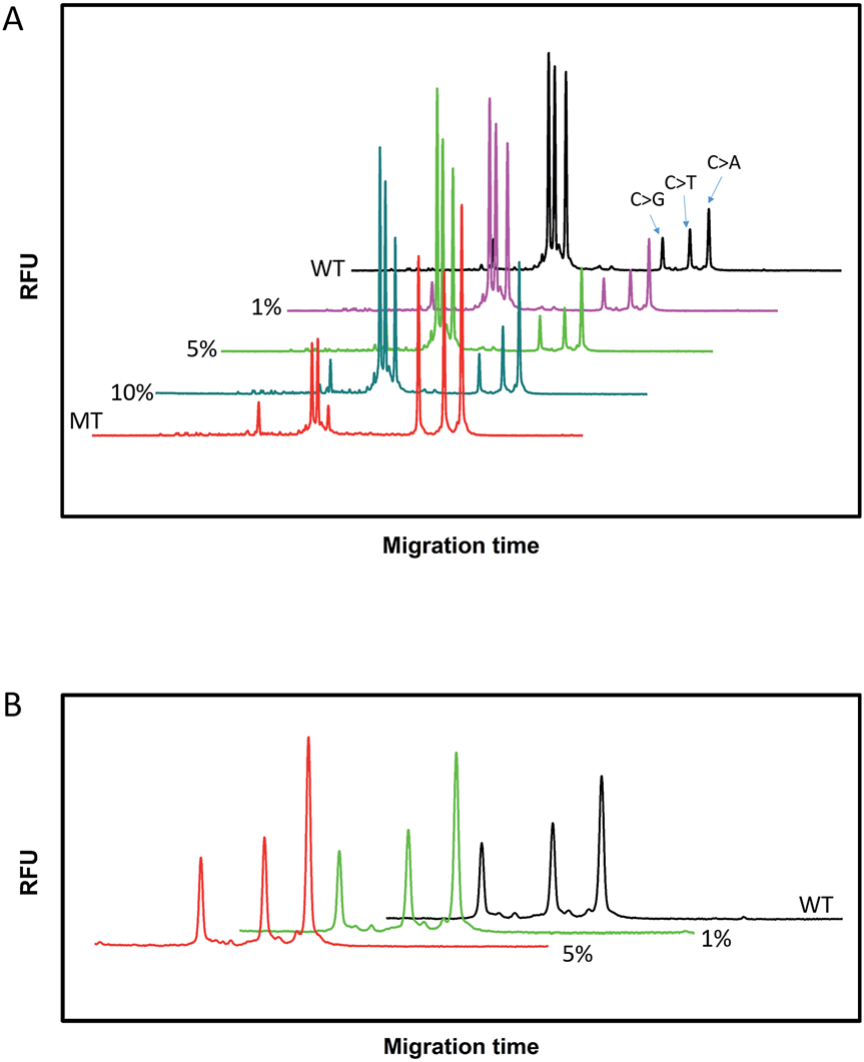
The electropherograms for the multiplexed SNP discrimination in the detection of STK11 gene (C > G, C > T, and C > A) using the CuAAC-based assay at different abundances (A), and at low abundances (0–5%) in an enlarged scale (B). WT means the tested sequences are all wild type targets. MT means the tested sequences are all mutant type targets.

To assess the multiplexing performance of this assay for SNP detection, the STK11 gene, which is associated with Peutz–Jeghers syndrome,^53^,^54^ was used as the detection target. Peutz–Jeghers syndrome is a kind of dominant genetic disease. The early diagnosis and prognosis of the disease can improve the quality of life of and reduce the mortality of patients. Since electrophoresis has the excellent ability to separate different lengths of oligonucleotides, a CuAAC-based assay can achieve multiplex detection only by simply encoding the DNA probes with different lengths. Three specific probes for the STK11 gene, rs59912467C > G(WT_1_/MT_1_), rs184528337C > T(WT_2_/MT_2_), and rs587778695C > A(WT_3_/MT_3_), were designed. The lengths of the DNA probes were adjusted with random deoxynucleotide, which has no interference with the hybridization between the DNA probes and the targets. Accordingly, the total length of the FAM-labeled P_M1_ and P_N1_ is 20 nucleotides (nts) for target MT_1_, P_M2_P_N2_ is 24 nts for target MT_2_, and P_M3_P_N3_ is 26 nts for target MT_3_. The all mutant type targets were mixed with wild type targets at abundances of 0% (the tested sequences were all wild type targets), 1%, 5%, 10% and 100% (the tested sequences were all mutant type targets). Then the sample mixtures were detected using the CuAAC-based assay in one-tube using the mixed specific probes. As shown in Fig. 4, the different lengths of the FAM-labeled products can be well separated and clearly detected. More importantly, the different mutant type targets can still be identified at an abundance of as low as 1% even under the complex conditions, which is better than those reported by some other methods for multiple SNP detection.^45^,^48^,^55^–^57^ Therefore, the CuAAC-based assay can be well used for the multiplex detection of SNPs.

## Conclusions

In summary, we have established a novel method for SNP discrimination using CuAAC-based assays. The enzyme-free click chemical ligation between N_3_-DNA and CH≡C-DNA makes the assay simple and robust without the need for special separation and purification. The SNP can be sensitively discriminated and the mutant type target can be identified at an abundance of as low as 0.5% in the presence of a wild type target. Moreover, the multiplexed analysis of SNP detection can be easily realized by simply encoding DNA probes of different lengths with the CuAAC-based assays. Therefore, we believe that this CuAAC-based SNP assay has great potential for clinical application.

## Conflicts of interest

There are no conflicts to declare.

## Supplementary Material

Supplementary information
