# Supplementary material for: Simultaneous multiple single nucleotide polymorphism detection based on click chemistry combined with DNA-encoded probes
Source: Chem Sci. 2018 Feb 22;9(13):3335–40. doi: 10.1039/c8sc00307f (PMC5932596; doi:10.1039/c8sc00307f)
Supplement: Supplementary file 1 [file SC-009-C8SC00307F-s001.pdf]

Electronic Supplementary Material (ESI) for Chemical Science.
This journal is © The Royal Society of Chemistry 2018

Electronic Supplementary Information

Simultaneous multiple single nucleotide polymorphism detection based on click chemistry combining with DNA-encoded probes

Qian-Yu Zhou, Fang Yuan, Xiao-Hui Zhang, Ying-Lin Zhou*, Xin-Xiang Zhang*
Beijing National Laboratory for Molecular Sciences (BNLMS), MOE Key Laboratory of Bioorganic Chemistry and Molecular Engineering, College of Chemistry, Peking University, Beijing 100871, China. E-mail: zhouyl@pku.edu.cn, zxx@pku.edu.cn; Fax: +86-10-62754112; Tel: +86-10-62754112.

Experimental methods

Materials and apparatus

$CuSO_4{\cdot}5H_2O$, $NaH_2PO_4{\cdot}2H_2O$, $Na_2HPO_4{\cdot}12H_2O$ and NaCl were purchased from Xilong Chemical Co., Ltd. (Guangdong, China). Sodium ascorbate was bought from Aladdin (Shanghai, China). Tris(3-hydroxypropyltriazolylmethyl)amine (THPTA) was obtained from Sigma-Aldrich (St. Louis, MO, USA) and stored at 4℃. HPLC purified oligonucleotides were obtained from Shanghai Sangon Biotech (Shanghai, China). The sequences and modifications used in this study are listed in Table S1 and S2. Deionized water was used in all experiments. All chemical reagents in the experiment were of analytical grade. Beckman coulter ssDNA 100-R Kit was acquired from Beckman (USA). The CuAAC reaction was performed on a C1000 Touch^{TM} PCR (USA). HPLC - ESI - MS was carried out on Dionex Ultimate 3000 UHPLC and Thermo Q - Exactive mass spectromer (Thermo Fisher, MA, USA). CuAAC products were separated and detected with a Beckman coulter PA800 plus (USA) quipped with an LIF detector (excitation at 488 nm)

The CuAAC reaction for target ssDNA detection

Probe P_M (50 nM) and probe P_N (50 nM) were mixed with different concentration of target DNA in 10 mM potassium phosphate solution (PBS, pH 7.4). The resulting solutions were heated to 85℃ for 5 min and then gradually cooled down to room temperature. Then a mixture of the catalyst solutions (2 mM THPTA, 100 μM $CuSO_4$, 1 mM sodium ascorbate) was added to the above solutions and the CuAAC reaction was carried out at room temperature for 2 h.

The CuAAC reaction for SNP detection

A certain ratio of probe P_M and probe P_N was mixed with an appropriate amount of target DNA or mutant DNA in PBS. The resulting solutions were heated to 85℃ for 5 min and then gradually cooled down to 30℃. Then a mixture of the catalyst solutions (2 mM THPTA, 100 μM $CuSO_4$, 1 mM sodium ascorbate) was added to the above solutions and the CuAAC reaction was carried out at 30℃ for 0.5 h.

The detection of the CuAAC products

The CuAAC products were mixed with 1 μL FAM labeled internal standard chain (IS) to correct the fluorescence intensity caused by aberration of sample injection, and subsequently injected into capillaries with application of a voltage of 9.3 kV for 2 s. The separation and detection of the CuAAC products were performed on the Beckman coulter PA800 plus with an electrophoresis voltage of 9.3 kV at 30℃ for an hour of running time. The positions and areas of the peaks were determined by the 32 Karat Software (Beckman). The relative peak area (RPA) which was used for the quantification of the CuAAC product was defined as the ratio of the peak area of CuAAC product

to the peak area of IS.

For HPLC-ESI-MS detection, elution was performed using 8 mM dimethylhexylamine and acetate acid (160 µL in 500 mL solution) as mobile phase A, and acetonitrile as mobile phase B for HPLC-MS. The injection volume was 20 µl. Scan range: 350 m/z to 4000 m/z. Mass resolution: 70 000.

Table S1. The oligonucleotides used in the exploring performance of SNP discrimination experiments (5′-3′)

Name	Sequence
P_M	FAM-TAGCTTATCAG-Azide
P_N	Alkyne-ACTGATGTTGA
Perfectly matched target (T)	TCAACATCAGTCTGATAAGCTA
Mismatch-1-T (N_1=T) target (M1)	TCAACATCAGTTTGATAAGCTA
Mismatch-2-C (N_2=C) target (M2)	TCAACATCAGTCCGATAAGCTA
Mismatch-3-T (N_3=C) target (M3)	TCAACATCAGTCTCATAAGCTA
Mismatch-4-A (N_3=T) target (M4)	TCAACATCAGTCTTATAAGCTA
Mismatch-5-C (N_3=A) target (M5)	TCAACATCAGTCTAATAAGCTA
Mismatch-6-G (N_4=G) target (M6)	TCAACATCAGTCTGGTAAGCTA
Mismatch-7-C (N_4=C) target (M7)	TCAACATCAGTCTGCTAAGCTA
IS	FAM-CTCG

Note: The underlined bases are mutant bases.

Table S2. The oligonucleotides used in the detection of STK11 rs59912467(C>G), rs184528337 (C>T) and rs587778695 (C>A) mutations (5′-3′)

Name	Sequence
P_{M1}(C>G)	FAM-CGATGTCCAA-Azide
P_{N1}(C>G)	Alkyne-GAGGTCCTCG
WT_1(C>G)	ACGAGGACCTCTTCGACATCGA
MT_1(C>G)	ACGAGGACCTCTTGGACATCGA
P_{M2}(C>T)	FAM-CGAGTCTCACT-Azide
P_{N2}(C>T)	Alkyne-GTGTCGCCCGCCG
WT_2(C>T)	GGGCGACACAGCGAGACTC
MT_2(C>T)	GGGCGACACAGTGAGACTC
P_{M3}(C>A)	FAM-CGTCCTGATTG-Azide
P_{N3}(C>A)	Alkyne-TAGATGATGTCATCC
WT_3(C>A)	GATGACATCATCTACACTCAGGAC
MT_3(C>A)	GATGACATCATCTACAATCAGGAC

Note: The underlined bases are mutant bases.

Table S3. Effect of mismatched position for SNP detection

	Relative peak area 1 (RPA1)	Relative peak area 2 (RPA2)	Ratio of RPA1 to RPA2
N_4=G：N_1=T （G-T）：（T-G）	2.329	2.997	0.777
N_4=C：N_3=T （T-C）：（C-T）	0.5353	0.4366	1.226
N_3=A：N_2=C （A-C）：（C-A）	0.4565	1.320	0.346

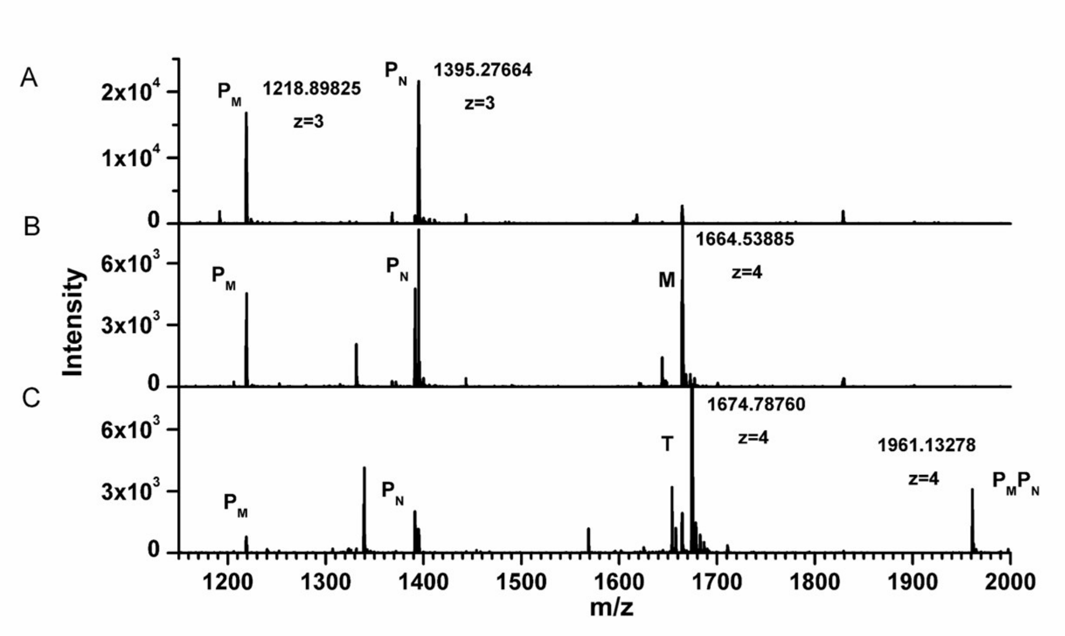

Fig. S1 Mass spectra of the template-directed CuAAC chemical reaction (A) without target (50 nM P_M +50 nM P_N), (B) with mismatched target M3 (50 nM P_M +50 nM P_N+100 nM M3), (C) with matched target T (50 nM P_M +50 nM P_N+100 nM T).

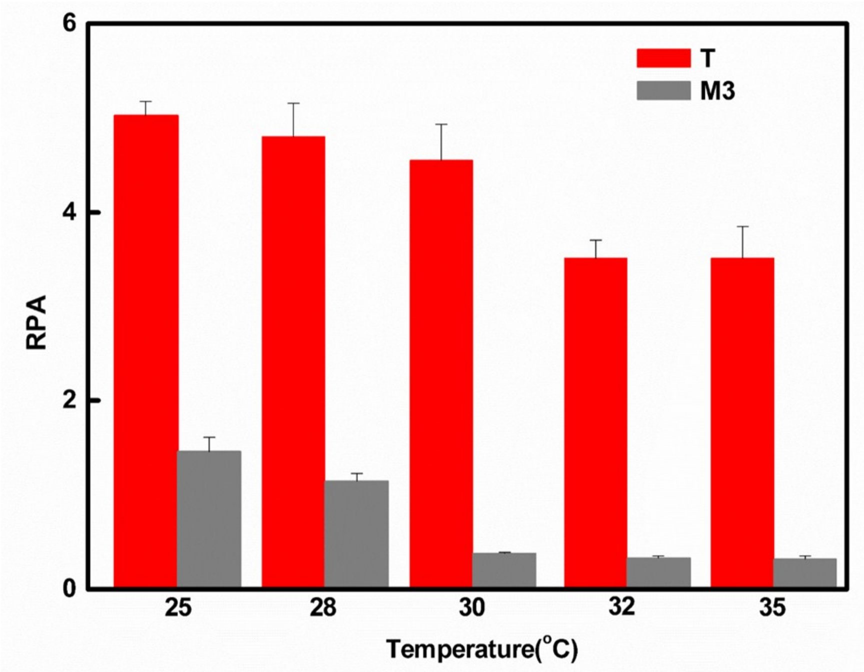

Fig. S2 The effect of temperature on SNP detection

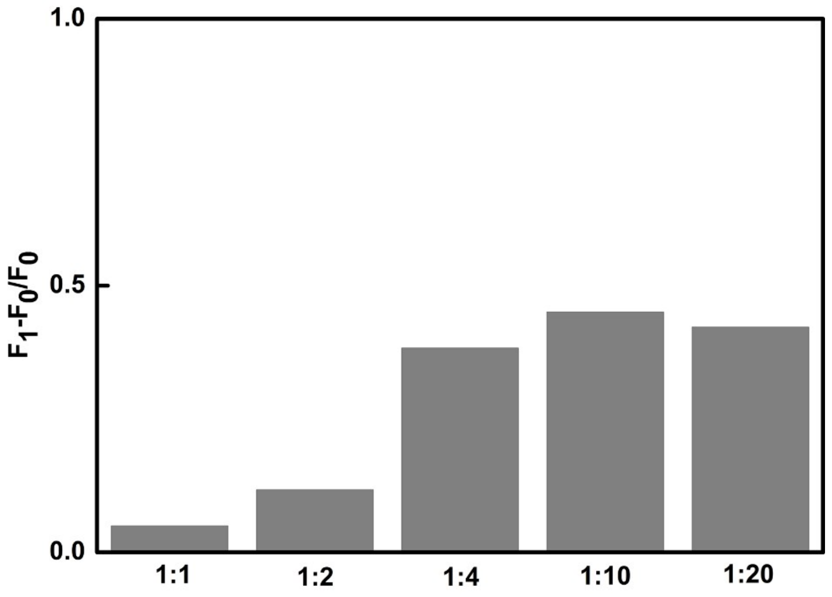

Fig. S3 The effect of ratios (P1:P2) on SNP detection. F_1 and F_0 represent the peak area produced by abundance 0.5%, and 100%, respectively.

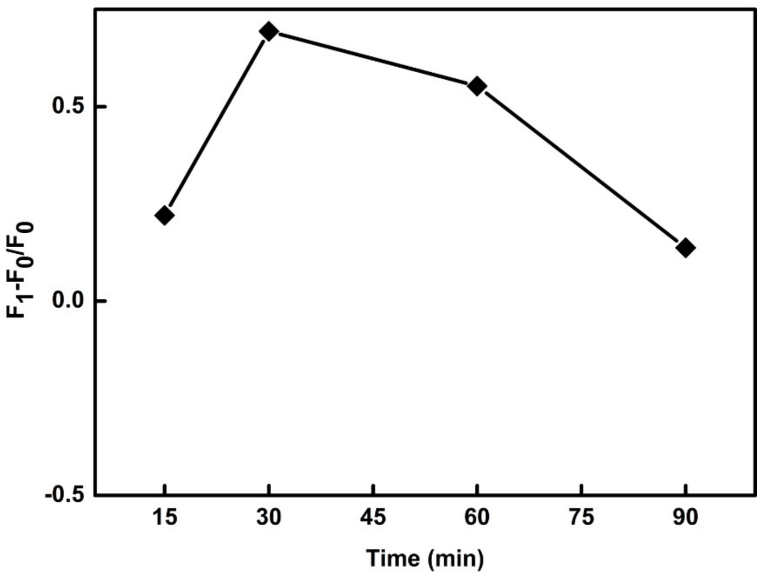

Fig. S4 The effect of reaction time on SNP detection. F_1 and F_0 represent the peak area produced by abundance 0.5%, and 100%, respectively.
